# Editorial Comment to Interstitial lung disease induced by apalutamide therapy for castration‐resistant prostate cancer: A report of a rare case

**DOI:** 10.1002/iju5.12428

**Published:** 2022-03-08

**Authors:** Takeshi Yuasa

**Affiliations:** ^1^ Department of Urology Cancer Institute Hospital Japanese Foundation for Cancer Research Tokyo Japan

Apalutamide, enzalutamide, and abiraterone acetate, which are new androgen receptor axis targeted (ARAT) agents, are currently available to treat metastatic hormone sensitive prostate cancer (HSPC).^1^, ^2^ Due to their excellent efficacy, they are rapidly being introduced in clinical practice in Japan, which is dramatically changing the therapeutic strategy for metastatic HSPC.^2^ Major adverse events for these agents, which include fatigue, hypertension, and bone loss, seem to be mild and manageable.^2^ Serious adverse events, especially interstitial lung disease (ILD), which is often caused by various anti‐cancerous agents, seems to be rarely caused by these ARAT agents.^2^, ^3^

In this issue of *IJU Case Report,* Kirishima et al. reported the case of a patient with possible apalutamide‐induced ILD.^3^ The patient presented with dyspnea with diffuse bilateral interstitial infiltrates and ground‐glass opacities in the upper and lower lobes of the lungs.^3^ Infectious diseases, including the novel coronavirus disease 2019 (COVID‐19), were ruled out and no other suspect drug was present, and thus, the patient was diagnosed with apalutamide‐induced ILD.^3^ The patient recovered after apalutamide discontinuation and intravenous steroid therapy with methylprednisolone (0.5 mg/kg/day).^3^

During anti‐neoplastic therapy, ILD is one of the most serious events because it hampers the anticancer therapy. The incidence of ILD is higher in Asian patients than in Western patients.^4^ When ILD is suspected, any drug that can possibly induce lung injury is usually discontinued promptly, regardless of its severity, and anti‐neoplastic therapy is not resumed until the patient’s lung injury has improved.^5^ Additionally, due to the increased risk of perioperative respiratory complications, post‐chemotherapy surgery may be impossible.^5^ Furthermore, due to the COVID‐19 pandemic, all patients who have respiratory distress and/or bilateral ground‐glass opacities on imaging studies need to have COVID‐19 ruled out. Excluding COVID‐19 infection may cause a delay in the final diagnosis and steroid administration. To obtain the optimal approach for ILD patients during anti‐neoplastic therapy, a multidisciplinary approach with a strategy that is carefully formulated by oncologists, pulmonologists, and infectious disease specialists should be recommended.

## Conflict of interest

The authors declare no conflict of interest.
